# Network pharmacology-based strategy to investigate the effect and mechanism of α-solanine against glioma

**DOI:** 10.1186/s12906-023-04215-1

**Published:** 2023-10-21

**Authors:** ChunPeng Wang, XiaoHui Liu, ShiWen Guo

**Affiliations:** 1 Department of Neurosurgery, The First Affiliated Hospital of Xi’an Jiaotong University, Xi’an, 710000 China; 2 Department of Medical Oncology, Anyang Cancer Hospital, An Yang, 455000 China

**Keywords:** α-Solanine, Network pharmacology, Glioma, STAT1

## Abstract

**Background:**

An anti-tumour activity has been demonstrated for α-solanine, a bioactive compound extracted from the traditional Chinese herb *Solanum nigrum L*. However, its efficacy in the treatment of gliomas and the underlying mechanisms remain unclear. The aim of this study was to investigate the inhibitory effects of α-solanine on glioma and elucidate its mechanisms and targets using network pharmacology, molecular docking, and molecular biology experiments.

**Methods:**

Traditional Chinese Medicine Systems Pharmacology Database and Analysis Platform (TCMSP) was utilized to predict the potential targets of α-solanine. GeneCards was used to gather glioma-related targets, and the STRING online database was used to analyze protein–protein interaction (PPI) networks for the shared targets. Hub genes were identified from the resulting PPI network and further investigated using Gene Ontology (GO) enrichment and Kyoto Encyclopedia of Genes and Genomes (KEGG) pathway analysis. Additionally, prognostic and gene set enrichment analyses (GSEA) were carried out to identify potential therapeutic targets and their underlying mechanisms of action in relation to the prognosis of gliomas. In vitro experiments were conducted to verify the findings from the network pharmacology analysis.

**Results:**

A total of 289 α-solanine targets and 1149 glioma-related targets were screened, of which 78 were common targets. 11 hub genes were obtained, including SRC, HRAS, HSP90AA1, IGF1, MAPK1, MAPK14, KDR, STAT1, JAK2, MAP2K1, and IGF1R. The GO and KEGG pathway analyses unveiled that α-solanine was strongly associated with several signaling pathways, including positive regulation of MAP kinase activity and PI3K-Akt. Moreover, α-solanine (10 µM and 15 µM) inhibited the proliferation and migration but promoted the apoptosis of glioma cells. Finally, STAT1 was identified as a potential mediator of the effect of α-solanine on glioma prognosis.

**Conclusion:**

α-Solanine can inhibit the proliferation and migration of gliomas by regulating multiple targets and signalling pathways. These findings lay the foundation for the creation of innovative clinical anti-glioma agents.

**Supplementary Information:**

The online version contains supplementary material available at 10.1186/s12906-023-04215-1.

## Introduction

Glioma, a primary tumor most frequently in the central nervous system (CNS), has become a significant global public health concern owing to its high mortality rate [1]. Although numerous contemporary treatments are available for glioma, it continues to be one of the most severe afflictions of the human nervous system, marked by a poor prognosis, a propensity for recurrence, and a significant occurrence of disability [2]. If left untreated, patients usually have a survival period of only a few months. As a result, there is a pressing requirement to explore innovative and effective medicinal treatments to combat or manage gliomas.

α-Solanine is a glycoalkaloid that occurs naturally and is synthesized by different plants, including *Solanum nigrum L.* (Longkui), a kind of traditional Chinese medicine (TCM), as well as several edible plants such as potatoes, cherries, and tomatoes [3]. Numerous studies have indicated that α-solanine offers a multitude of health benefits, including anti-inflammatory properties and the capacity to boost the immune system [4, 5]. It has also been reported to exhibit anti-cancer activity against different types of tumors, such as those present in the breast, liver, pancreas, colon, and other regions [6]. Although the potential of α-solanine for treating glioma has been investigated, its precise mechanism of action remains unclear.

**Fig. 1:**
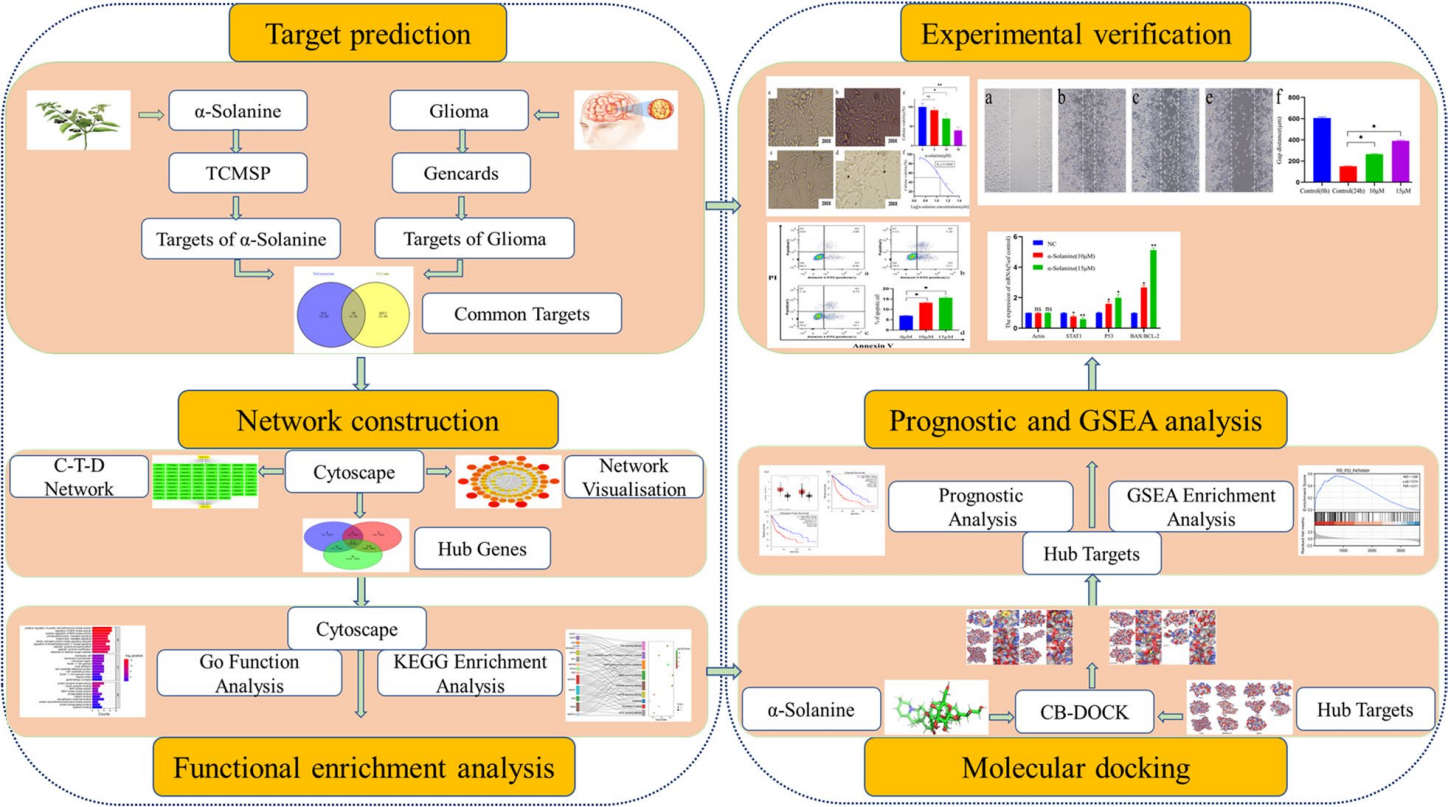
The flowchart of this study

Network pharmacology is a rapidly evolving field of research that aims to unravel the intricacies of disease and drug mechanisms within complex biological networks [7]. This approach has revolutionized investigations by providing a systematic framework to address scientific challenges at multiple levels. In recent years, there has been a growing scholarly emphasis on network pharmacology research with the objective of utilizing Traditional Chinese Medicine (TCM) for the management of tumors. This surge in interest arises from the methodology's capacity to discern regulators possessing multi-targeting capabilities and synergistic effects, thereby elucidating the fundamental mechanisms of TCM in a manner that aligns with the holistic principles of the traditional medical system [8–10]. In particular, the accuracy and reliability of this approach deserve recognition [11]. In the present study, we employed a network pharmacology method to determine potential targets and underlying mechanisms by which α-solanine inhibits glioma. The results were subsequently validated through molecular docking and biological experiments. A diagram of the presented workflow is shown in Fig. 1.

## Materials and methods

### Acquisition target genes of α-solanine and glioma

To identify genes targeted by α-solanine in glioma, we conducted a keyword search for "α-solanine" using the TCMSP (http://tcmspw.com/). To obtain genes related to glioma, we used the GeneCards database (https://www.genecards.org) and searched for the keyword "glioma".

### Identifying the common targets between drugs and disease

To identify the common targets between α-solanine and glioma, we applied the intersection method and represented the common targets using a Venn diagram. Furthermore, we constructed a network using Cytoscape v3.6.1 to visualize the connections between the component, common targets and diseases.

### Construction of PPI network

The intersection targets were integrated into the STRING database (https://string-db.org/) to obtain information on their protein–protein interaction (PPI) network [12]. We established the screening parameters for the organism as "Homo sapiens" with a minimum required interaction score of "highest confidence (0.9)". Subsequently, the resulting PPI information was visualized using Cytoscape, and a PPI network was constructed.

Cytoscape can calculate various parameters of individual nodes in network diagrams, including degree centrality (DC), betweenness centrality (BC), closeness centrality (CC), and average shortest path length (ASPL), which facilitate in-depth analysis of the characteristics of nodes in an interaction network [13]. In this study, the identification of hub targets for the treatment of glioma with α-solanine was accomplished by selecting nodes that exhibited DC, BC, and CC values exceeding the corresponding median values in the PPI network.

### GO and KEGG enrichment analysis

Gene Ontology (GO) enrichment analysis is a common method for identifying biological processes, molecular functions, and cellular components. Meanwhile, the Kyoto Encyclopedia of Genes and Genomes (KEGG) pathway enrichment analysis can reveal significant signalling pathways associated with biological processes. In this study, we conducted GO and KEGG enrichment analyses of the hub targets identified using the DAVID database (https://DAVID.ncifcrf.gov) [14]. The results were further visualized using Bioinformatics software (https://www.bioinformatics.com.cn).

### Molecular docking analysis

The application of molecular docking has become essential in computational drug design [15]. In the study, we employed molecular docking to investigate the mode and strength for the interaction between α-solanine and the hub targets. To perform a docking analysis, we adopted CB-Dock (http://cao.labshare.cn/cb-dock/), a recently developed blind docking technology that can autonomously identify the sites for binding to a specific protein and conduct molecular docking using AutoDock [16]. CB-Dock has been proven to surpass other cutting-edge blind docking techniques in accurately forecasting binding locations and binding structures [17]. The crystal structures of the hub protein targets were downloaded from the Protein Data Bank. (http://www.rcsb.org). For the ligand, the 3D structure of α-solanine was retrieved from the PubChem Compound database (https://www.ncbi.nlm.nih.gov). The protein (receptor) and α-solanine (ligand) files were then submitted to the CB-Dock website for molecular docking analysis.

### Prognostic and GSEA analysis of hub targets

To reveal the prognostic value of α-solanine in individuals with glioma, we conducted survival analysis on its hub targets. The hub targets were entered into the GEPIA database (http://gepia.cancer-pku.cn) to screen for potential therapeutic targets relevant to the prognosis of glioma patients [18].

To investigate the mechanisms associated with the differential expression of therapeutic targets in glioma and gain insight into how α-solanine affects the glioma prognosis, we used GSEA software (version 3.0) to examine the biological functions related to the differences in the expression of therapeutic targets between normal and glioma tissues. The reference gene sets were based on the c2 sets of the Molecular Signatures Database (KEGG gene sets,c2.all.v6.2.symbols.gmt), and the permutation number was set at 1,000. The gene sets enriched through GSEA with a nominal *P*-value below 0.05 and a false discovery rate (FDR) below 0.25 were deemed to be of statistical significance [19].

## Cell culture

The human glioma-derived U87MG cell line, a typical grade IV glioma cell line, was purchased from Wuhan Pu-nuo-sai Life Technology Co. Ltd. in Wuhan, China. This particular cell line is renowned for its vigorous proliferation and tumorigenic properties [20]. The cells were cultivated in RPMI-1640 medium supplemented with 10% fetal bovine serum, 100 µg/mL ampicillin, and 100 µg/mL streptomycin.

### Cell morphology visualization

Stock solutions of α-solanine (1 mmol/l) were prepared in dimethyl sulfoxide (DMSO) and diluted with double-distilled water containing 10% DMSO to obtain various concentrations. To investigate the effects of α-solanine on U87MG cells, three different concentrations of the compound (5 µM, 10 µM and 15 µM) were applied to the cells for a period of 24 h. The resulting morphological changes in the cells were then analyzed using a light microscope (Leica, Germany).

### Cell viability assay

To evaluate the impact of α-solanine on cell viability, U87MG cells were cultured in 96-well plates at a density of 4 × 10^3^ cells per well and subjected to treatment with α-solanine at varying concentrations of 5 µM, 10 µM and 15 µM for 24 h. The Cell Counting Kit-8 (CCK-8) assay was performed to measure cell proliferation, with absorbance measurements recorded at a wavelength of 450 nm. GraphPad Prism 7.0 software was then used for data analysis and plotting.

### Scratch test

U87MG cells were cultured in 6-well plates at a density of 3 × 10^5^ cells per well, allowing them to form a monolayer. To create a wound, three lines were drawn vertically in each well using a sterile pipette aspirator. The cells were then treated with a drug-free medium (serum-free) or a drug-containing medium (serum-free with 10 µM or 15 µM α-solanine) for 24 h. Following treatment, the cells were imaged using a light microscope (Leica, Germany), and the distance between the markers on the cells was measured at both 0 and 24 h. The relative migration distance was calculated using the formula: Relative migration distance (mm) = distance within the scratch at 0 h—distance within the scratch at 24 h. Each experiment was performed in triplicate.

### Flow cytometry analysis for apoptosis

U87MG cells were subjected to treatment with α-solanine at concentrations of 10 and 15μΜ for 48 h after being cultured in 6-well plates at 37 °C. To determine the extent of apoptosis, the Annexin V-FITC early apoptosis kit (Shanghai Yeasen Biotechnology Co., Ltd., Shanghai, China) and a flow cytometer (Guava Technologies, Hayward, CA, USA) were used. After washing the treated cells with cold phosphate-buffered saline, they were resuspended in 400 μL of 1 × binding buffer, and 5 μL annexin V-FITC and 5 μL PI staining solution were added. After incubation in the dark, flow cytometry analysis was performed within one hour.

### Quantitative Polymerase Chain Reaction (qPCR) assay

The RNA Easy Fast Cell Kit (Tiangen, China) was used to extract total RNA from U87MG cells according to the manufacturer's instructions. The quality of the RNA was evaluated using a SpectraMax Quick Drop reader (Molecular Devices, USA). Reverse transcription was performed using the iScript cDNA synthesis kit (Bio-Rad) on 1 μg of RNA. RT-qPCR was conducted using SYBR Green Real-Time PCR Master Mix (Toyobo, Japan) to measure the mRNA expression levels of four genes: STAT1, P53, BAX, and BCL-2. The β-actin gene served as the internal control, and the expression of each gene was calculated using the 2^−ΔΔCt^ method. The primer sequences used for qPCR analysis are listed in Additional file 1.

### Statistical analysis

Continuous data that followed a normal distribution were represented as the mean value ± the standard deviation (SD). GraphPad Prism 7.0 software was used for conducting statistical analysis, and the two-tailed unpaired Student's t-test was used to determine differences between the groups. Statistical significance was indicated by an asterisk (*: *P* < 0.05) or two asterisks (**: *P* < 0.01).

## Results

### Identifying gene targets for α-Solanine in Glioma

**Fig. 2:**
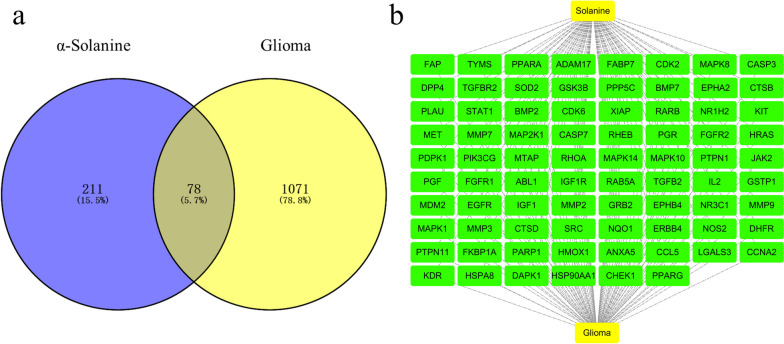
Overlapping target genes between glioma and α-solanine. **a** The Venn diagram shows the targets of α-solanine and those related to gliomas. **b** Cytoscape visualizes 78 of the common targets in the PPI network

Based on the TCMSP database, we identified 290 genes that are targeted by α-solanine. To ensure consistency in gene names and species information, we standardized the data using the UniProt database, leading to a final set of 289 genes. Simultaneously, 1149 disease-related genes were obtained from the GeneCards database (Additional file 2). The two datasets were merged for co-analysis, resulting in the identification of 78 overlapping genes, representing 5.7% of the total genes analyzed, as shown in Fig. 2a.

### Construction of Compound-Target-Disease (CTD) network

The pharmacological potential of α-solanine for glioma treatment was visualized using the Cytoscape software to construct a network of α-solanine-common targets-glioma (Fig. 2b). The network consists of 78 nodes representing common target genes (depicted in green) and two supplemental nodes (depicted in yellow) corresponding to α-solanine and glioma. The network diagram showed 155 edges representing the connections between the nodes. The findings indicate that α-solanine exerts a inhibitory effect on glioma cell proliferation by acting on multiple targets.

### Construction and analysis of PPI network

**Fig. 3:**
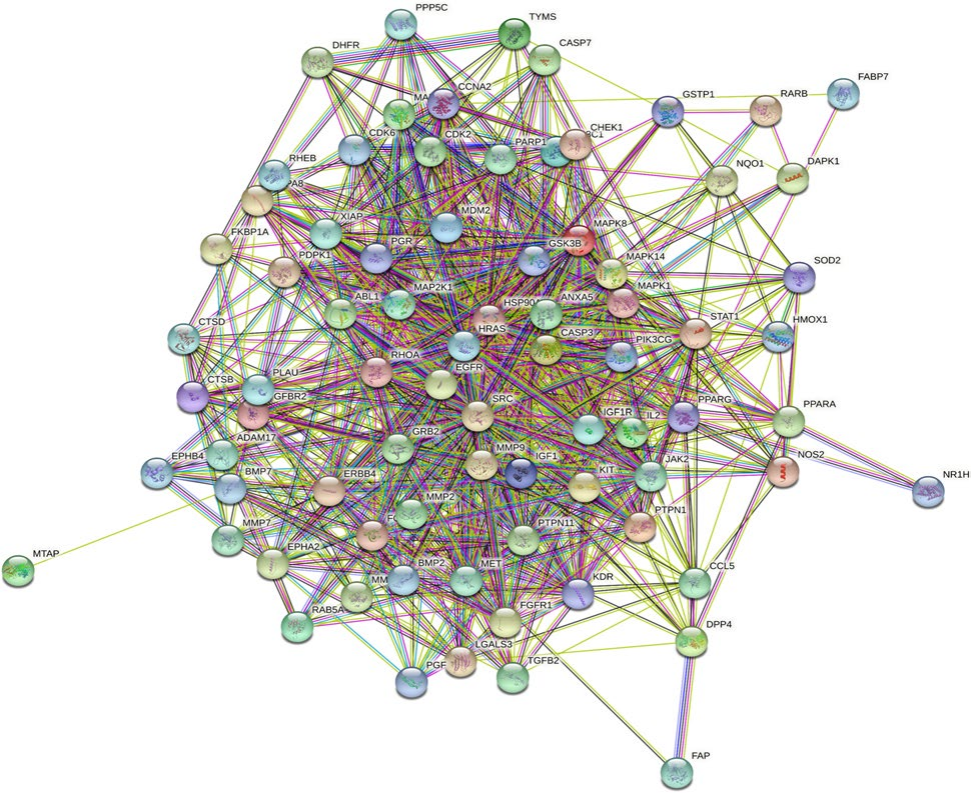
PPI network of common targets generated by STRING

**Fig. 4:**
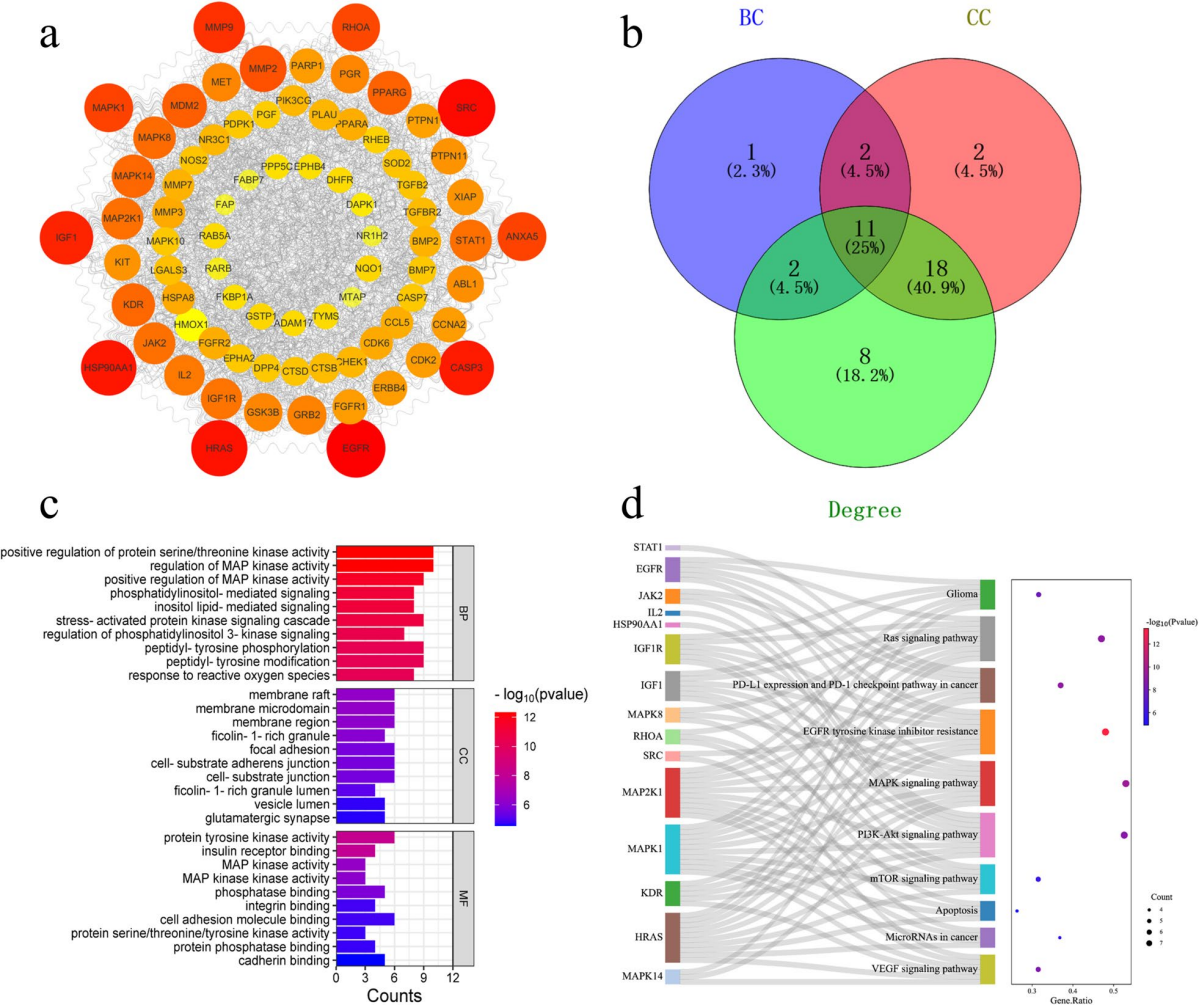
The extraction and functional enrichment analysis of hub genes. **a** After visualising the nodes, bigger sizes and colours from red to yellow refer to higher degree values. **b** Wayne diagram displaying the hub genes. **c** The top 10 GO terms of hub genes. **d** The top 10 enriched pathways of hub genes [21]

PPI analysis was performed for the 78 genes commonly targeted by α-solanine based on data in the STRING database (Fig. 3). The resulting TSV data were imported into Cytoscape, which generated a network diagram consisting of 78 nodes and 885 edges, as presented in Fig. 4a. The size and colour intensity are directly proportional to the degree value and target probability. Finally, 11 hub targets were identified based on their DC, BC, and CC values (Fig. 4b and Table 1). A list of attribute values for the remaining 67 common targets is included in Additional file 3.

**Table 1:** The hub targets information of PPI network

Name	Degree	BC(Betweenness Centrality)	CC(Closeness Centrality)
SRC	56	0.047796203	0.777777778
HRAS	54	0.058248009	0.762376238
HSP90AA1	53	0.058715751	0.762376238
IGF1	50	0.036957342	0.740384615
MAPK1	43	0.03418052	0.681415929
MAPK14	35	0.021703121	0.636363636
KDR	35	0.050558721	0.641666667
STAT1	33	0.027782546	0.62601626
JAK2	33	0.056614531	0.631147541
MAP2K1	33	0.010874876	0.62601626
IGF1R	32	0.019065703	0.636363636

### GO and KEGG pathway enrichment analysis

A total of 1680 GO terms were enriched, with 1525 belonging to BP, 37 to CC, and 118 to MF. The enriched BP terms were predominantly related to the regulation of phosphatidylinositol 3-kinase signalling, response to reactive oxygen species, and positive regulation of MAP kinase activity. The CC terms were mainly related to membrane raft, membrane microdomain, and membrane region. Moreover, the MF category had enriched terms in protein tyrosine kinase activity, MAP kinase activity, integrin binding, and protein phosphatase binding. The top 10 GO terms ranked by their adjusted* p*-values are displayed in Fig. 4c. The terms with lower adjusted *p*-values are coloured red, and this indicate higher enrichment.

KEGG pathway analysis revealed 119 pathways, comprising 11 disease signalling pathways and 39 molecular signalling pathways among the top 50 enriched KEGG pathways. Figure 4d demonstrates the top 10 disease signalling pathways with high counts, including the PI3K-Akt signalling pathway, mTOR signalling pathway, apoptosis, microRNAs in cancer, glioma, PD-L1 expression, and PD-1 checkpoint pathway in cancer. Our findings suggest that α-solanine may exert its therapeutic effects on glioma through various potential biological mechanisms.

### Verification of compound‑target interaction

**Fig. 5:**
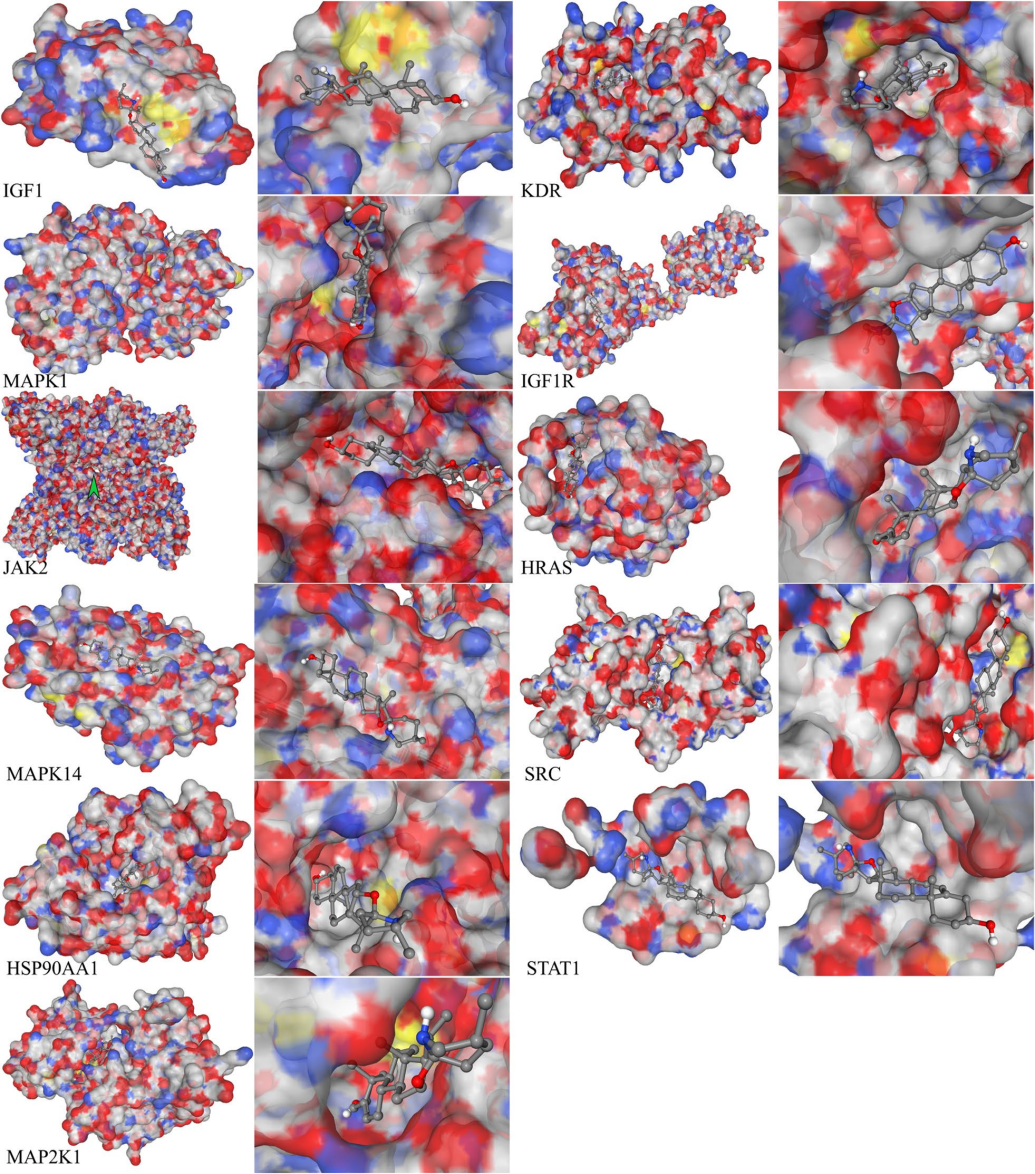
Docking results of α-solanine with the hub protein targets. The PubChem substance ID of α-Solanine is 348,289,415. The protein structures of IGF1 (PDB ID: 8EYR), KDR (PDB ID: 1WQ9), MAPK1 (PDB ID: 6G54), IGF1R (PDB ID: 7XLC), JAK2 (PDB ID: 3EYG), HRAS (PDB ID: 8CXF), MAPK14 (PDB ID: 5ETC), SRC (PDB ID: 1KC2), HSP90AA1 (PDB ID: 5NJX), STAT1 (PDB ID: 3VNE), and MAP2K1 (PDB ID: 5HZE) were collected from the PDB database

We selected 11 hub target proteins for molecular docking to verify the interaction between α-solanine and its potential targets. The structure of α-solanine was then uploaded to CB-Dock to analyse its potential for docking with SRC, HRAS, HSP90AA1, IGF1, MAPK1, MAPK14, KDR, STAT1, JAK2, MAP2K1, and IGF1R. The docking scores for each target protein were documented in Table 2. According to the Vina scoring system, a score of -5.0 kcal/mol or lower is indicative of favorable binding interactions between ligands and receptors [22]. Therefore, the molecular docking analysis revealed a significant affinity between α-solanine and the 11 hub target proteins. These findings not only validate the accuracy of the selected hub targets but also suggest the potential effectiveness of α-solanine in glioma treatment. The docking sketches of target proteins with α-solanine are presented in Fig. 5 and Additional file 4.

**Table 2:** The binding energy of compound and hub targets (kcal/mol)

Receptors	PDB	Vina	Cavity		Center			Size	
	ID	score	size	X	Y	Z	X	Y	Z
IGF1	1B9G	-16.4	67	19	2	-19	24	24	24
KDR	1VR2	-10.3	634	19	12	10	24	24	24
MAPK1	1GOL	-10.1	596	67	12	6	24	24	24
IGF1R	1IGR	-9.6	891	21	1	62	24	24	24
JAK2	2B7A	-9.4	2168	248	-57	37	30	24	24
HRAS	121P	-9.3	1215	-5	-9	-7	24	24	24
MAPK14	1A9U	-8.9	260	-9	28	60	24	24	24
SRC	1A07	-8.6	1209	42	12	28	24	24	24
HSP90AA1	1BYQ	-8.5	705	40	-48	65	24	24	24
STAT1	1BF5	-8.3	313	4	13	31	24	24	24
MAP2K1	1S9J	-8	2981	35	32	40	32	24	24

To display the active site crystal structure of the protein, we utilized a color-coding scheme where carbon atoms are represented by white, oxygen atoms by red, nitrogen atoms by blue, and sulfur atoms by yellow. Meanwhile, the crystal pose of the ligand is depicted using a different color scheme, where hydrogen atoms are white, carbon atoms are grey, and oxygen atoms are red.

### Survival analysis and GSEA analysis of the key targets

**Fig. 6:**
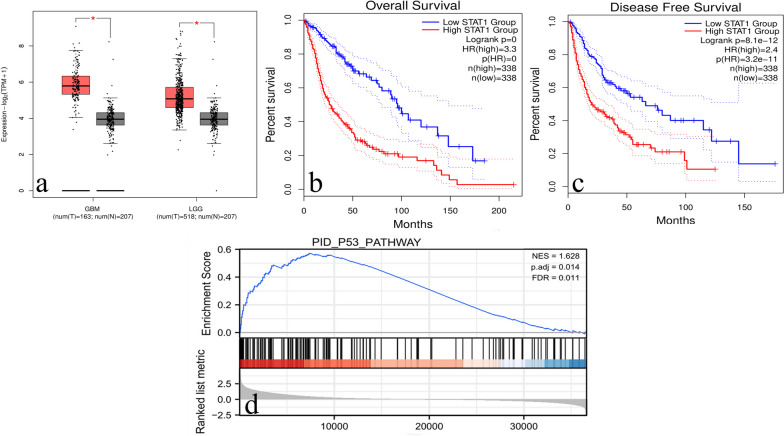
The expression levels analysis, survival analysis and GSEA of STAT1. **a** STAT1 was overexpressed in high- grade glioma tissues (*n* = 518) and low-grade glioma tissues (*n* = 163), compared with normal brain tissues(*n* = 207). Red boxes represent tumor tissues; Gray boxes represent normal brain tissues; ∗ *P* < 0.05. **b**, **c** STAT1 overexpression was associated with a worse prognosis in glioma patients. **d** The results of GSEA showed that the p53 signaling pathway was enriched in normal samples with low STAT1 expression

An investigation was conducted utilizing data from the GEPIA database to examine the expression and prognostic relevance of 11 hub targets in glioma. It was observed that there was a significant increase in STAT1 mRNA transcription in LGG and GBM tissues compared to normal tissues, according to our findings. Moreover, STAT1 was the only hub target significantly correlated with the overall survival (OS) and disease-free survival (DFS) of glioma patients (Fig. 6a-c).

To better understand how α-solanine exerts its therapeutic effects through the hub target STAT1 and improves the prognosis of glioma patients, we conducted a GSEA analysis to identify potential biological signalling pathways affected by the overexpression of STAT1 in glioma. As depicted in Fig. 6d, our results revealed an inverse correlation between STAT1 overexpression in glioma cells and the p53 pathway, which has been identified as a beneficial factor for enhancing the overall outlook of patients with glioma [23].

### α-Solanine suppressed the proliferation of glioma cells

**Fig. 7:**
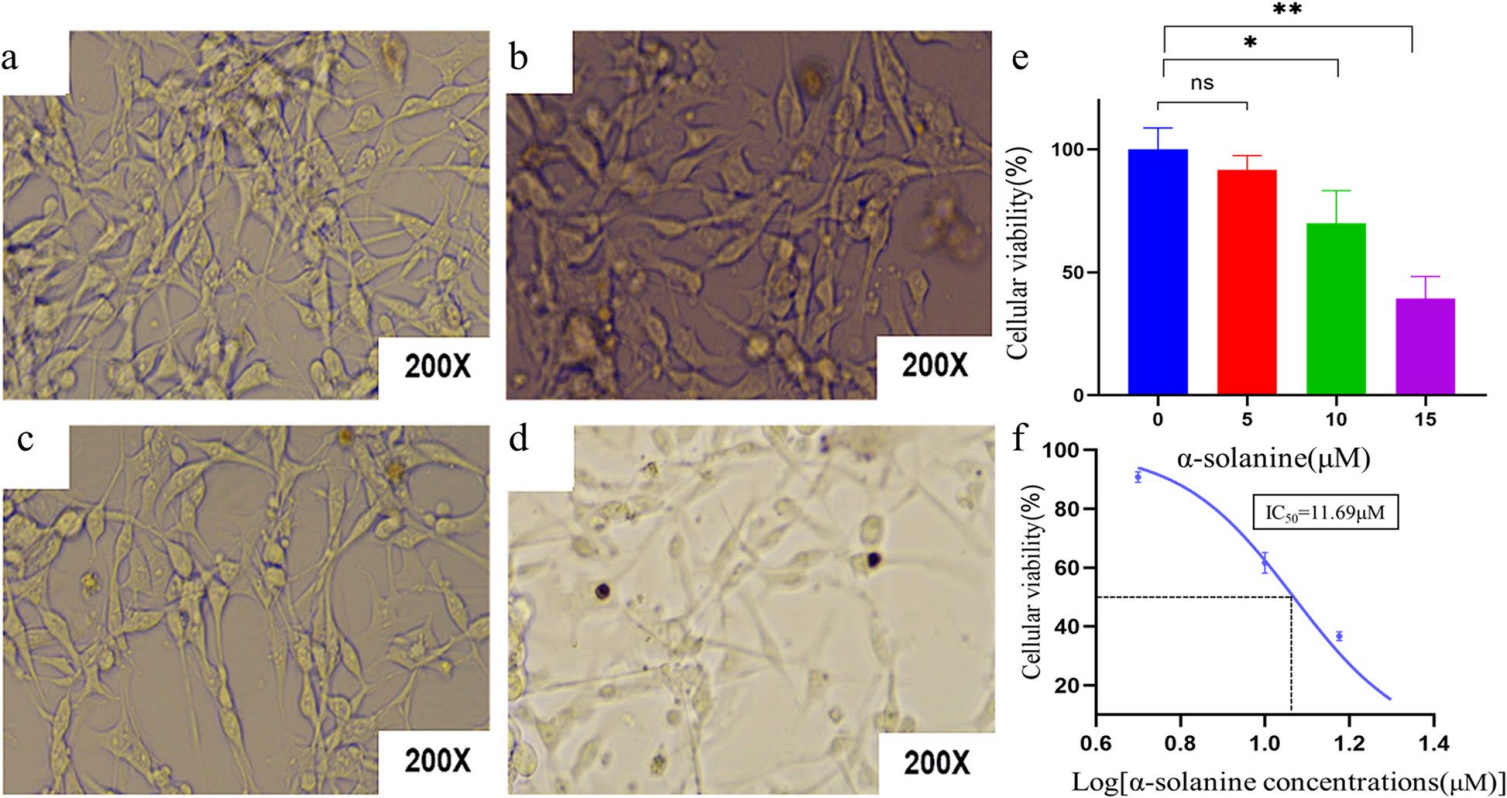
Effects of α-solanine on the proliferation of U87MG cells. **a**-**d** Morphological changes of U87MG cells treated with 0, 5, 10, and 15 µM of α-solanine for 24 h, respectively. **e** α-solanine inhibited the viability of U87MG cells as measured by the CCK8 assay. Data are presented as mean ± SD for three independent experiments. **f** Dose–response curve in the CCK8 assay of U87MG cells treated with α-solanine, yielding a calculated IC50 of 11.69 µM. *P < 0.05, ** *P* < 0.01, ns:not significant

In order to investigate the effects of α-solanine on glioma cell proliferation, U87MG cells were treated with various concentrations of α-solanine. The morphology of cells was examined using an optical microscope. It was observed that α-solanine had no significant effect on the viability of U87MG cells when administered at a concentration of 5 µM (Fig. 7a–b). Consequently, only the concentrations of 10 µM and 15 µM were employed in all subsequent experimental procedures. At a concentration of 10 µM, we observed a reduction in the number of glioma cells, an increase in cell spacing, and a change in cell shape towards an oval and contracted morphology. When the α-solanine concentration was further increased to 15 µM, we observed pronounced cell wall wrinkling and nuclear consolidation, accompanied by a marked decrease in the glioma cell count and the appearance of turbidity in the cell culture medium (Fig. 7c-d).

Further statistical analysis showed that concentrations of 10 and 15 μM significantly inhibited the proliferation of glioma cells (Fig. 7e). Nonlinear regression analysis of the data obtained from the CCK-8 experiments was then performed (GraphPad Prism 7.0) to obtain the IC_50_ valuesof α-solanine against glioma cells (Fig. 7f). Overall, these results suggested that α-solanine suppresses the proliferation of glioma cells in a dose-dependent manner.

### α-Solanine inhibited migration of glioma cells

**Fig. 8:**
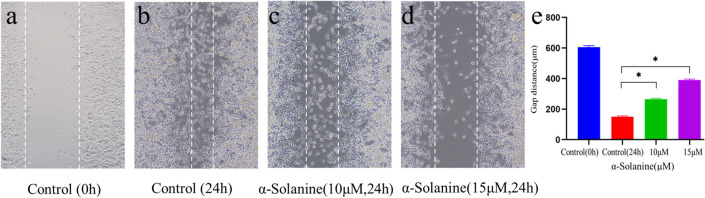
α-Solanine inhibited migration and invasion of U87MG cells in vitro. **a**-**d** Cell migration U87MG was tested with a wound-healing assay. Photographs (100 × magnification) show representativemigration in scraped areas after 24 h incubation without (control) or with the indicated concentrations of α-solanine.. The error bars represent the standard error (*n* = 3). * *P* < 0.05

In vitro cell scratching experiments were conducted to investigate the effect of α-solanine on the migratory ability of U87MG cells. The results, including the initial scratch displacement for each cohort, as well as the inter-group scratch distance after 24 h with or without α-solanine treatment, are shown in Fig. 8. We calculated the relative migration distances of glioma cells in each group and compared the findings between the α-solanine treatment and the control group. The results of the statistical analysis clearly revealed that α-solanine concentrations of 10 µM and 15 µM effectively decreased the relative migration distances of glioma cells.

### α-Solanine promoted apoptosis of glioma cells

**Fig. 9:**
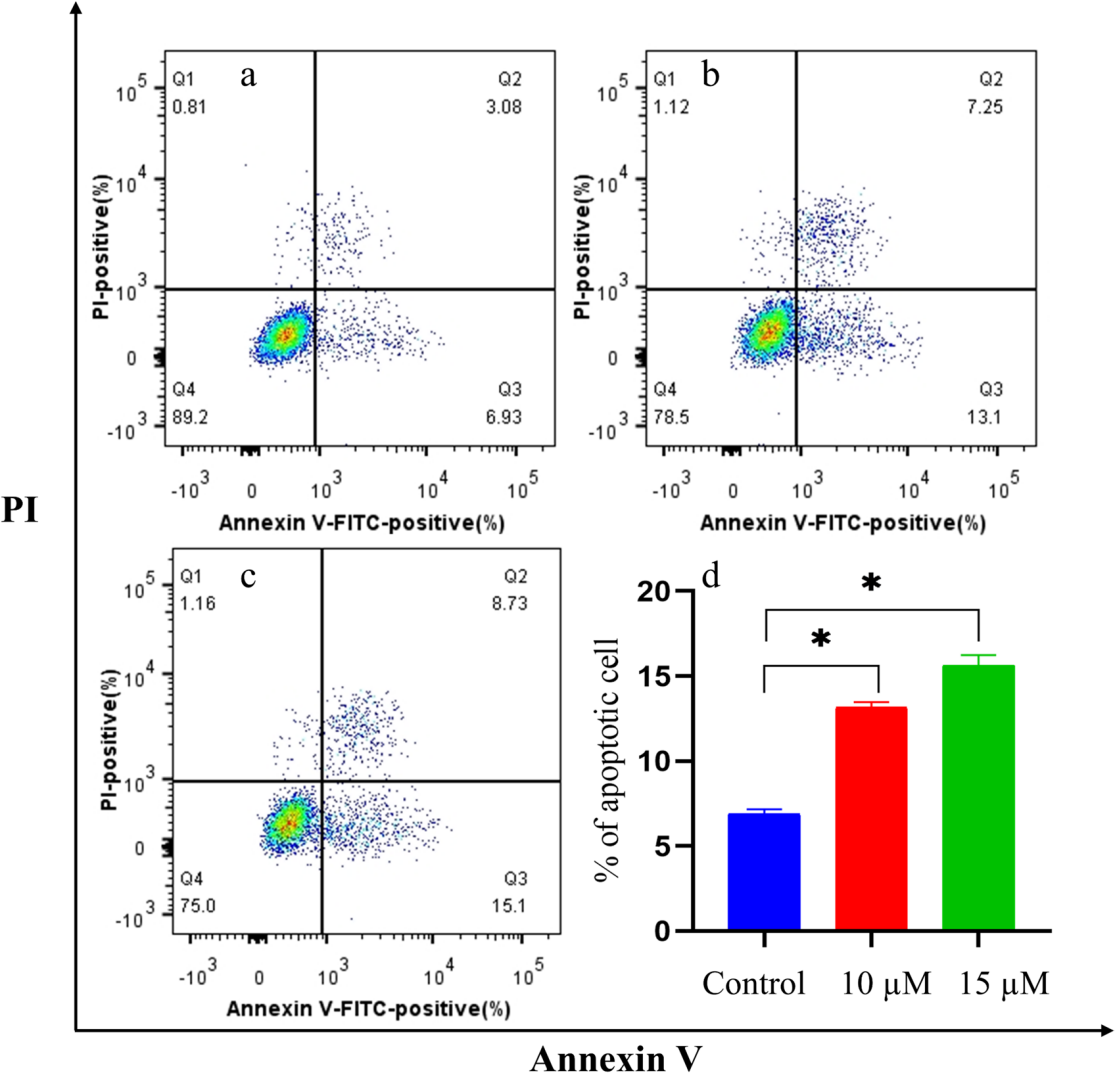
α-Solanine promoted the apoptosis of U87MG cells. **a**-**c** Ratios of total apoptosis and early apoptosis in U87MG cells treated with α-solanine at concentrations of 0 µM,10 µM,15 µM, respectively, were examined based on flow cytometric detection. **d** The histogram shows the percentage of early apoptotic cells in different treatment groups. The error bars represent the standard error (*n* = 3). * *P* < 0.05

The effect of α-solanine on apoptosis in U87MG cells was investigated using flow cytometry. Our results showed that treatment with α-solanine at concentrations of 10 µM and 15 µM significantly increased early apoptosis (Fig. 9), demonstrating the efficacy of α-solanine in inducing apoptosis of U87MG cells.

### Effect of α-solanine on mRNA expression levels of STAT1, P53, BAX, and BCL-2 in glioma cells

**Fig. 10:**
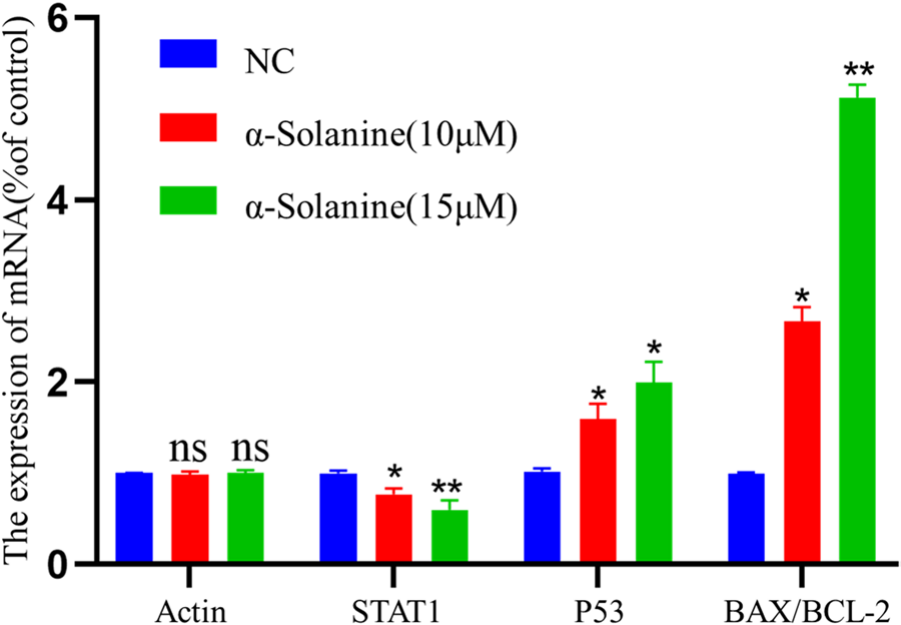
The mRNA expressions of Actin, STAT1, P53 and Bax/Bcl-2 in the control group(NC), and effects of α-solanine on the mRNA expression of these genes by RT-PCR in U87MG cells. The error bars represent standard error (*n* = 3). * *P* < 0.05, ** *P* < 0.01, ns:not significant

Real-time PCR analysis was performed on U87MG cells to validate the GSEA analysis results. Treatment with α-solanine significantly decreased the transcription of STAT1mRNA, but markedly enhanced P53mRNA expression levels (Fig. 10). In addition, we observed a considerable increase in the ratio of Bax mRNA to Bcl-2 mRNA after α-solanine treatment.

## Discussion

Glioma is a highly aggressive neoplasm that originates from glial cells, with a poor prognosis, and it commonly affects the brain and spinal cord. Standard treatment modalities for glioma include surgical resection, chemotherapy, and radiation therapy [24]. Despite these options, a significant proportion of patients experience recurrence within 6–10 months of surgical treatment, resulting in an overall 5-year survival rate of less than 3% [25]. Recent evidence indicates that even with standardized treatment, including surgery, chemotherapy, and radiation therapy, the 5-year survival rate for glioma patients remains below 5% [26], which has led to the notion that gliomas are incurable [27].

Traditional Chinese Medicine (TCM) has a long history of application in treating various diseases and is now considered a complementary or alternative approach to conventional medicine [28]. In contemporary times, it has witnessed significant advancements, leading to an increase in the use of herbal medications to treat tumors [29]. *Solanum ni grum Linn*. (Longkui) is a widely used traditional Chinese medicinal herb with anticancer properties. Its main steroidal glycoalkaloid, α-solanine, is highly effective against various types of cancer in all three stages: carcinogenesis, growth, and metastasis [4, 30, 31]. These studies have offered valuable indications for detecting the correlation between α-solanine and glioma. In this study, we applied network pharmacology to identify the possible targets of α-solanine and elucidate its underlying mechanisms against glioma.

The present study employed a systematic approach to identify potential target genes for α-solanine treatment of glioma based on relevant databases and Venn diagrams. Subsequently, 78 candidate genes were identified, and their interactions were visualized using protein–protein interaction (PPI) analysis. Through further network analysis using Cytoscape,11 hub target genes, including SRC, HRAS, HSP90AA1,IGF1, MAPK1, MAPK14, KDR, STAT1, JAK2, MAP2K1, and IGF1R, were selected for subsequent investigation. Molecular docking analysis was performed to determine whether α-solanine binds with the hub targets. The results revealed that α-solanine exhibited high binding affinity with the 11 core target genes, suggesting its potential as a therapeutic agent for glioma.

SRCs are critical members of the SRC family of protein tyrosine kinases and play a crucial role in various cellular processes, including morphology, motility, proliferation, and survival [32]. Inhibiting SRC and upregulating PTEN significantly reduces the migration and invasion of glioma stem cells [33], leading to a growing interest in developing novel SRC inhibitors for glioma treatment [34]. Additionally, the expression levels of HRAS, MAPK1, IGF1, and IGF1R in glioma cells have been closely linked to the growth, invasion, and metastasis of glioma [35–38]. Previous studies have identified MAPK14 (p38) and KDR as potential tumour suppressors in glioma development and potential antigens for vaccine advancement [39, 40]. HSP90AA1 is consistent with MAPK14 in playing a vital role in regulating apoptosis [41]. Therefore, targeting these hub targets may have important implications for the treatment of glioma.

The GO analysis revealed that α-solanine participates in several critical biological processes in glioma, including the regulation of reactive oxygen species (ROS) response, mitogen-activated protein (MAP) kinase activity, and phosphatidylinositol 3-kinase (PI3K) signalling, among others. ROS plays a crucial role in inducing apoptosis of tumour cells, and various medicinal plants have been found to increase ROS levels to induce cancer cell apoptosis [3, 42, 43]. Moreover, both MAP kinase activity and PI3K signalling are associated with the mitogenic process [44], and the cessation of mitosis can trigger apoptosis in tumorigenic cells [45].

The KEGG pathway analysis suggested that α-solanine inhibits the proliferation of glioma through several pathways, such as the Ras signalling pathway, PD-L1 expression and the PD-1 checkpoint pathway, the PI3K-Akt signalling pathway, MAPK signalling pathways, and VEGF signalling pathways. The Ras signalling pathway is an essential component of cellular signalling pathways and plays a crucial role in oncogenic signalling [46]. The PD-1 and PD-L1 pathways serve as a crucial checkpoint in the immune system, regulating its activity and preventing over activation. Recent studies have shown that blocking these pathways could suppress the progression of several cancers [47]. The PI3K-Akt pathway has been the subject of extensive research in tumorigenesis and has been implicated in various aspects of glioma development, including formation, migration, invasion, and apoptosis [48]. Studies have also demonstrated that Girdin, an actin-binding protein, regulates the migration and invasion of glioma cells through the PI3K-Akt signalling pathway [49]. Additionally, the signalling pathways of MAPK and VEGF have crucial functions in the processes of glioma cell migration, proliferation, and angiogenesis [50, 51].

STAT1, as a transcription factor, plays a significant role in cytokine-mediated signalling pathways that have a range of biological effects such as antiviral activities, suppression of tumour cell growth, and promotion of apoptosis [52]. Although STAT1 is commonly regarded as a tumour suppressor, several studies have highlighted its oncogenic role in the pathogenesis of tumours [53, 54]. A recent study reported that STAT1 has a protumorigenic role in gliomas [55]. Additionally, high expression of STAT family members predicts poor glioma prognosis [56]. In agreement with these studies, our findings showed that STAT1 was upregulated in glioma specimens compared with matched normal tissue specimens, and its high expression was associated with poor OS and DFS in glioma patients, supporting its potential as a reliable prognostic indicator and a promising therapeutic target for glioma.

Despite the well-established knowledge of solanine's inhibitory effects on multiple signaling pathways such as NF-κB, ERK1/2, AKT and STAT1, its precise effect on gliomas through STAT1 remains uncertain [57]. According to the GSEA results, STAT1 was found to negatively regulate the p53 pathway in glioma cells. Two in vitro experiments conducted on human fibrosarcoma cells and lymphocytes confirmed this finding that STAT1 in tumor cells has a regulatory effect on the p53 pathway, demonstrating that the overexpression of STAT1 suppressed MDM2 and upregulated P53 expression [58, 59]. We hypothesized that α-solanine could decrease STAT1 expression in glioma cells, which, in turn, could activate the p53 signalling pathway. Subsequently, our qRT-PCR results validated this hypothesis, which showed a significant decrease in STAT1 expression and a substantial increase in P53 expression with an increase in α-solanine concentration. Furthermore, considering that the Bax/Bcl-2 pathway is a pro-apoptotic pathway positively regulated by P53 [60, 61], the significant increase in the Bax/Bcl-2 ratio following α-solanine intervention indicates that p53 and its downstream signaling pathways were activated, inducing U87MG cell apoptosis. Therefore, we conclude that α-solanine enhances the activity of the P53 signalling pathway by down-regulating STAT1 expression, initiating apoptosis of glioma cells, and ultimately contributing to a favorable prognosis for glioma patients.

In recent years, network pharmacology, which combines principles from systems and computational biology, has emerged as a valuable research tool for investigating the network regulatory effects of TCM or formulas in the context of various diseases [14]. Molecular docking is another powerful tool that can validate the accuracy of network pharmacology results and predict biological experiments by simulating the interaction between small molecules and biological macromolecules [62, 63]. Therefore, the amalgamation of network pharmacology and molecular docking trials can amplify the efficacy of TCM investigation. In this study, we also applied experimental methods to explore the possible mechanisms of α-solanine against glioma at the molecular and cellular levels, complementing the results of network pharmacology. However, our research has some limitations. First, some potential α-solanine targets may have been overlooked due to the inherent challenges in obtaining relevant information from the Network Pharmacology database. Second, while GO and KEGG enrichment analysis predicted multiple signaling pathways targeted by α-solanine in glioma cells, the detailed mechanisms require further investigation. Third, although our findings provide a foundation for future research into the potential treatment of gliomas with α-solanine, further investigation are necessary to translate these findings into clinical applications.

## Conclusions

α-Solanine is a promising and safe TCM for the treatment of glioma. Our network pharmacological analysis revealed that α-solanine exerts an anti-glioma effect by modulating the expression or activity of multiple targets, pathways and biological processes crucial for cellular metabolism and apoptosis. Specifically, α-solanine improves the glioma prognosis by down-regulating the expression of STAT1. Further exploration of the potential benefits of α-solanine glioma treatment is warranted to validate our findings.

### Supplementary Information

**Additional file 1: Table S1.** PCR primers and their sequences used in this study.

**Additional file 2: Table S2. **Targets of disease and drug action and their intersection.

**Additional file 3.** The attribute values of 78 common targets.

**Additional file 4: Figure S1.** The key amino acid residues of STAT1interact with α-solanine. Blue dashed lines represent hydrogen bonds, gray dashed lines represent hydrophobic interactions, and yellow dashed lines represent salt bridges.

## Data Availability

The data in the current study come from the TCMSP database (https://tcmsp-e.com/) and the GeneCards database (https://www.genecards.org). The article and its additional files include all the data generated or analyzed in this study.
