# Supplementary material for: Network pharmacology-based strategy to investigate the effect and mechanism of α-solanine against glioma
Source: BMC Complement Med Ther. 2023 Oct 21;23:371. doi: 10.1186/s12906-023-04215-1 (PMC10589944; doi:10.1186/s12906-023-04215-1)
Supplement: Supplementary file 1 — Additional file 1: Table S1. PCR primers and their sequences used in this study. [file 12906_2023_4215_MOESM1_ESM.docx]

Table S1: PCR primers and their sequences used in this study.

| Primer names | Primer sequence |
| --- | --- |
| β-Actin | 5′- TCACCCACACTGTGCCCATCTACGA-3′ |
|  | 3′-GGTAACCGTTACTCGCCAAGGCGAC-5′ |
| STAT1 | 5′-GATCAGCTGCAAACGTGGTTC-3′ |
|  | 3′-AGGGAGTCGTCGAATTTTTCG-5′ |
| P53 | 5′- TAGTGTGGTGGTGCCCTATG -3′ |
|  | 3′-AGGGAGTCGTCGAATTTTTCG-5′ |
| BAX | 5′- ATGG AGGGGTCCGGGGAG -3′ |
|  | 3′- AGTCGG GTAG AAGA AGGT -5′ |
| BCL-2 | 5′- ATGTGTGTGGAGAGCGTCAA -3′ |
|  | 3′- AACTAAAGAGGCACGACCGACAGAG-5′ |
