# Supplementary material for: Network pharmacology-based strategy to investigate the effect and mechanism of α-solanine against glioma
Source: BMC Complement Med Ther. 2023 Oct 21;23:371. doi: 10.1186/s12906-023-04215-1 (PMC10589944; doi:10.1186/s12906-023-04215-1)
Supplement: Supplementary file 4 — Additional file 4: Figure S1. The key amino acid residues of STAT1interact with α-solanine. Blue dashed lines represent hydrogen bonds, gray dashed lines represent hydrophobic interactions, and yellow dashed lines represent salt bridges. [file 12906_2023_4215_MOESM4_ESM.pdf]

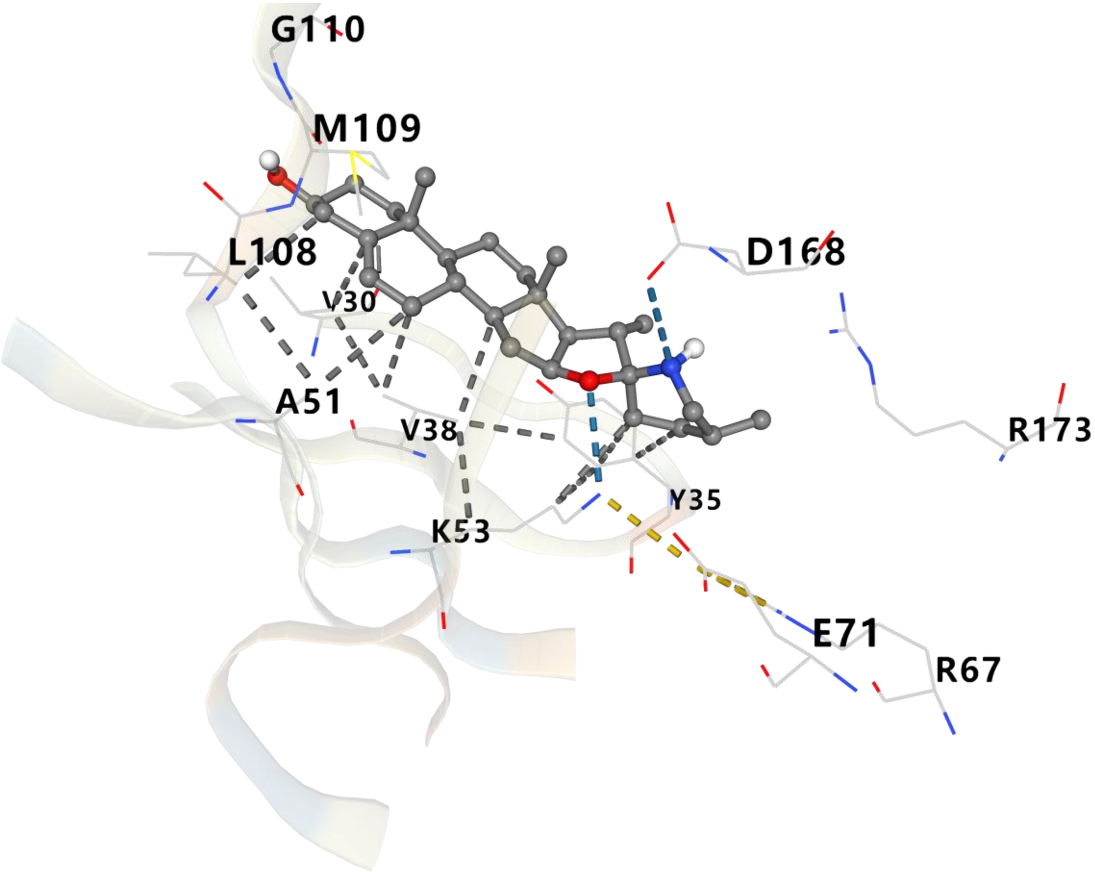

Figure S1: The key amino acid residues of STAT1 interact with α-solanine. Blue dashed lines represent hydrogen bonds, gray dashed lines represent hydrophobic interactions, and yellow dashed lines represent salt bridges.
